# Strain-specific estimation of epidemic success provides insights into the transmission dynamics of tuberculosis

**DOI:** 10.1038/srep45326

**Published:** 2017-03-28

**Authors:** Jean-Philippe Rasigade, Maxime Barbier, Oana Dumitrescu, Catherine Pichat, Gérard Carret, Anne-Sophie Ronnaux-Baron, Ghislaine Blasquez, Christine Godin-Benhaim, Sandrine Boisset, Anne Carricajo, Véronique Jacomo, Isabelle Fredenucci, Michèle Pérouse de Montclos, Jean-Pierre Flandrois, Florence Ader, Philip Supply, Gérard Lina, Thierry Wirth

**Affiliations:** 1 Institut de Systématique, Evolution, Biodiversité, UMR-CNRS 7205, Muséum National d’Histoire Naturelle, Université Pierre et Marie Curie, Ecole Pratique des Hautes Etudes, Sorbonne Universités, Paris, France; 2 Laboratoire Biologie Intégrative des Populations, Ecole Pratique des Hautes Etudes, PSL Research University, Paris, France; 3 Centre International de Recherche en Infectiologie, CIRI, University of Lyon, France; 4 Institut des Agents Infectieux, Hospices Civils de Lyon, Lyon, France; 5 Comité Départemental d’Hygiène Sociale, CLAT69, Lyon, France; 6 Agence Régionale de Santé Rhône-Alpes, Lyon, France; 7 Laboratoire de Bactériologie, Institut de Biologie et de Pathologie, CHU de Grenoble, Grenoble, France; 8 Laboratoire TIMC-IMAG, UMR 5525 CNRS-UJF, UFR de Médecine, Université Grenoble Alpes, Grenoble, France; 9 Laboratoire des Agents Infectieux et d’Hygiène, CHU de Saint-Etienne, Saint-Etienne, France; 10 Laboratoire Biomnis, Lyon, France; 11 Laboratoire de Biométrie et Biologie Evolutive, UMR CNRS 5558, University of Lyon, France; 12 Service des Maladies Infectieuses et Tropicales, Hôpital de la Croix-Rousse, Hospices Civils de Lyon, Lyon, France; 13 INSERM U1019, CNRS-UMR 8204, Center for Infection and Immunity of Lille, Institut Pasteur de Lille, Université de Lille, Lille, France

## Abstract

The transmission dynamics of tuberculosis involves complex interactions of socio-economic and, possibly, microbiological factors. We describe an analytical framework to infer factors of epidemic success based on the joint analysis of epidemiological, clinical and pathogen genetic data. We derive isolate-specific, genetic distance-based estimates of epidemic success, and we represent success-related time-dependent concepts, namely epidemicity and endemicity, by restricting analysis to specific time scales. The method is applied to analyze a surveillance-based cohort of 1,641 tuberculosis patients with minisatellite-based isolate genotypes. Known predictors of isolate endemicity (older age, native status) and epidemicity (younger age, sputum smear positivity) were identified with high confidence (*P* < 0.001). Long-term epidemic success also correlated with the ability of Euro-American and Beijing MTBC lineages to cause active pulmonary infection, independent of patient age and country of origin. Our results demonstrate how important insights into the transmission dynamics of tuberculosis can be gained from active surveillance data.

The tuberculosis (TB) agent *Mycobacterium tuberculosis* complex (MTBC) has plagued mankind for millennia and, in spite of important efforts to slow down its progression, will probably continue to do so for decades^1^,^2^. TB prevalence is highly contrasted between world regions. Most patients with TB live in low-income countries while prevalence can be very low in high-income countries. Such a prevalence contrast, along with increasing population movements and migrations, has led to a situation in which the TB epidemiology and the MTBC population structure in low-prevalence areas is nowadays strongly impacted by influx of TB patients originating from high-prevalence areas^3^,^4^,^5^,^6^. Even single events of exogenous strain introduction in a low prevalence area can lead to rapid epidemic spread and large TB transmission clusters after a certain period of time in certain contexts^7^,^8^.

Having the ability to capture the transmission dynamics and the epidemic success over time of particular strain groups from contemporary bacterial populations, and to identify associated contributions of pathogen- and/or host-related factors, could thus have important implications for epidemiological control and the understanding of bacterial evolution. In principle, past population dynamics of pathogens and the contribution of pathogen- or host-associated factors could be inferred from studies combining bacterial genetic data with patient clinical or socio-demographic data. Indeed, inferences based on population genetics methods and the coalescent theory, such as the skyline plot estimates of the evolution of population size over time^9^,^10^,^11^, have been successfully used by our group^12^,^13^ and others^14^,^15^,^16^ to detect important demographic events in MTBC history such as, for instance, episodes of strong expansion of the Beijing MTBC lineage during the Industrial Revolution and the First World War. However, current coalescent-based methods analyze correlates of epidemic success at broad strain group levels, such as species or lineages, rather than on individual strains^17^. Therefore, these methods inherently carry the risk of mixing strains with distinct demographic histories, potentially averaging out important strain-specific characteristics. Conversely, performing separate analyses on smaller groups of isolates substantially increases the uncertainty of the demographic estimates^18^.

In this work, we postulated that proxy measures of bacterial population dynamics such as epidemic success, endemicity and epidemicity, can be estimated at the level of each individual isolate in a study population. After demonstrating the relevance of this approach in simulations, we investigated a diversified MTBC population, typical of those seen in low TB prevalence areas^4^, obtained from a cohort of 1,641 TB patients from the Rhône-Alpes region of France. Our analysis discriminated isolates of epidemic strain groups introduced recently in the region from those of the regional endemic background. Finally, the inclusion of isolate-level estimates of epidemic success in regression-based association analyses identified both expected and novel links between MTBC transmission dynamics and the characteristics of patient and strain groups in our setting.

## Results

### Estimating epidemic success from genetic distances

Proposing a quantitative correlate of the epidemic success of a pathogen is difficult owing to the lack of a formal and consensual definition of epidemic success^19^. Here we define epidemic success as a purely quantitative and time-dependent concept: the epidemic success of a bacterial group is proportional to the frequency of its associated transmission events during a given period of time.

All else equal, and assuming a strain transmission rate that is higher than strain mutation rate (which is reasonable for TB)^20^, epidemic success in a successful group increases prevalence faster than diversity, resulting in a more clonal (i.e., less diverse) structure compared to other groups in the sample. Lower diversity results into smaller genetic distances between isolates. From a statistical standpoint, both the prevalence of, and pairwise genetic distances between isolates in a group can be jointly quantified by a measure of density in the space of genetic distances, suggesting that density correlates with success. Importantly, density is defined for all points in the space of genetic distances, hence on the level of individuals in the population. Based on this rationale, we postulated that a measure of density associated with the haplotype of an isolate reflects the epidemic success of its ancestors compared to other isolates in the sampled population.

**Figure 1:**
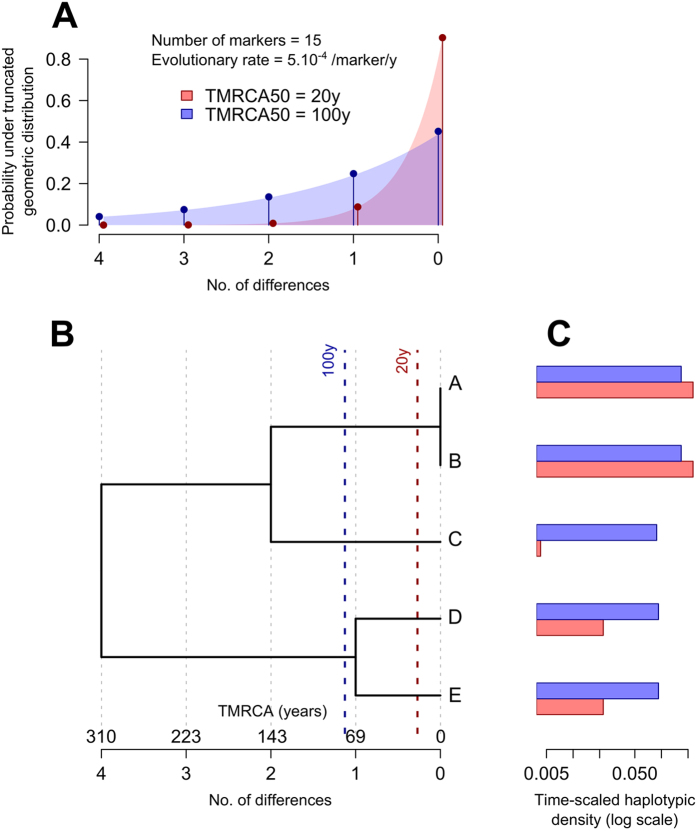
Time-scaled haplotypic density. THD computations were exemplified using a synthetic set of five 15-marker haplotypes (panel B). The timescales were defined as the median of a geometric distribution expressed in units of time (dashed lines in panel B; time units indicated above the X axis), based on the functional relationship between the genetic distance and the time to the most recent common ancestor (TMRCA; see Methods). Pairwise genetic distances were then associated with probabilities under the truncated geometric distribution (panel A). Probabilities decreased with the distance in a timescale-dependent fashion, with a faster decrease using the shorter 20y timescale (red curve) compared to the 100y timescale (blue curve). For each haplotype, THD was defined as the average of the probabilities associated with the distance from this haplotype to the others (panel C). Using a short timescale, haplotypes A and B, which have close relatives in the population, had much larger THDs compared to haplotype C, which has no close relative (red bars). Using a longer timescale, haplotype C had THD similar to that of haplotypes D and E because the densities of their respective clades were comparable relative to the timescale (blue bars). Remark the larger variance of the THD estimates with a short timescale compared to the larger timescale.

We constructed the density measure using an application-specific adaptation of a classical non-parametric technique, namely kernel density estimation (KDE)^21^. In the general case, KDE computes density based on distances between points and a kernel function, endowed with a bandwidth parameter to control the smoothness of the estimate. In our application, points were haplotypes, distances were the pairwise numbers of allelic differences and the kernel function was based on the geometric distribution. To control the bandwidth of the analysis in an interpretable fashion, we expressed this bandwith as a timescale parameter equal to the median time to the most recent common ancestor (TMRCA50, see Methods) under the kernel distribution and an evolutionary rate known a priori (Fig. 1). Intuitively, the timescale allows one to focus the analysis on recent transmission events (e.g. to detect epidemic isolates with short-term success) or to extend this focus towards the past (e.g. to detect endemic isolates with long-term success). In the following, we refer to KDE-based density estimates as timescaled haplotypic densities (THDs).

### Timescaled haplotypic density correlates with epidemic success in silico

**Figure 2:**
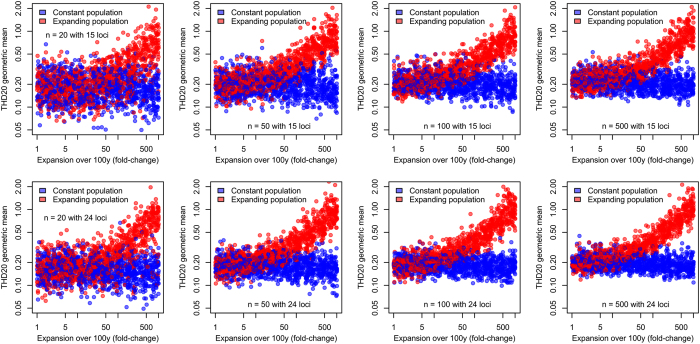
Timescaled haplotypic density (THD) of simulated constant-size and expanding populations. Markers represent scaled THD geometric means for 1,000 simulated metapopulations per panel, each comprising of a basal population with constant effective size (blue) and an epidemic population expanding with exponential growth over 100y (red) with varying expansion fold-change (X-axis), sample size per population and number of genetic loci.

To investigate how THD reflects expansion events (epidemic bursts), we generated synthetic sets of haplotypes by means of Fastsimcoal 2 software^22^. Model parameters were carefully selected to mimic MTBC populations with genotypes obtained from independent minisatellite loci (as is the case in our cohort), with an evolutionary rate μ = 5 × 10^−4^ change per locus per year, selected as the average of previous estimates^13^,^23^,^24^,^25^,^26^ ranging from μ = 10^−4^ to 10^−3^; a generation time of one day; and a contemporary effective population size *N*_0_ = 10^7^ as determined from our previous analysis of MTBC haplotypes obtained from minisatellite data^13^. To simulate the success of a pathogen population, we used a scenario in which independent epidemic subpopulations emerge from a constant-size (*N*_0_ = 10^7^) basal population and grow exponentially during 100y to reach the same contemporary population size as the basal population. Simulations used expansion factors up to 500-fold over 100y (~6% yearly increase), of the same order of magnitude as previous estimates of the expansion of successful MTBC clades^13^,^15^,^27^. Scaled geometric means of THDs per population (see Methods) with a 20y timescale and varying sample sizes, numbers of VNTR loci and fold-change expansions of the epidemic subpopulations are shown in Fig. 2. Additional simulations using 1 and 10 kbp DNA sequences in place of VNTRs are depicted in Supplementary Fig. S1. Collectively, these results demonstrate that: i) THD correlates with population expansion; ii) expectedly, estimation accuracy increases with sample size and, to a lesser extent, with the number of genetic loci; and iii) scaled THDs are invariant relative to the number of markers.

### Characteristics of MTBC-infected patients in the Rhône-Alpes region of France

We investigated a collection of MTBC isolates representative of the Rhône-Alpes region of France, a low-MTBC prevalence area^28^. A total of 1,641 unique MTBC isolates (i.e. all from different patients) were recovered from the database of the Observatoire Rhône-Alpin des Mycobactéries (ORAM), a regional network of healthcare institutions involved in tuberculosis diagnosis and surveillance, from 2008 to 2014. Based on surveillance data available for the year 2010, our cohort included approximately 55% of all newly diagnosed TB patients in the region (see Supplementary Methods). Available socio-demographic, clinical and microbiological data, including indications of the proportion of missing data, are summarized in Table 1. French-native patients accounted for one-third of cases, consistent with previous reports in similar low-prevalence settings^5^. Rates of multidrug-resistance and resistance to first-line antibiotics rifampicin and isoniazid were 3.7, 3.8 and 10.1%, respectively.

### MTBC population structure

Two classical complementary genotyping methods were performed on the 1,641 MTBC isolates included, namely spoligotyping^29^ and MIRU-VNTR typing with a standard 15-locus scheme^30^. Spoligotyping is based on the detection of a collection of unique spacer sequences in a CRISPR locus^29^,^31^. Spoligotypes can be compared to databases to assign the strain to a family, a sublineage or a lineage. MIRU-VNTR typing interrogates multiple genomic loci containing variable numbers of tandem repeats. Compared to spoligotyping, the resolution power of MIRU-VNTR typing for distinguishing MTBC strains is higher, and as such, it can be used as a proxy for inferring recent transmission of MTBC strains^12^. MIRU-VNTR typing is also more robust-although clearly imperfect compared to DNA sequences, due to homoplasy-than spoligotyping for phylogenetic classification^31^, and it has been used successfully to investigate population dynamics at the level of MTBC lineages^13^,^32^,^33^,^34^.

**Figure 3:**
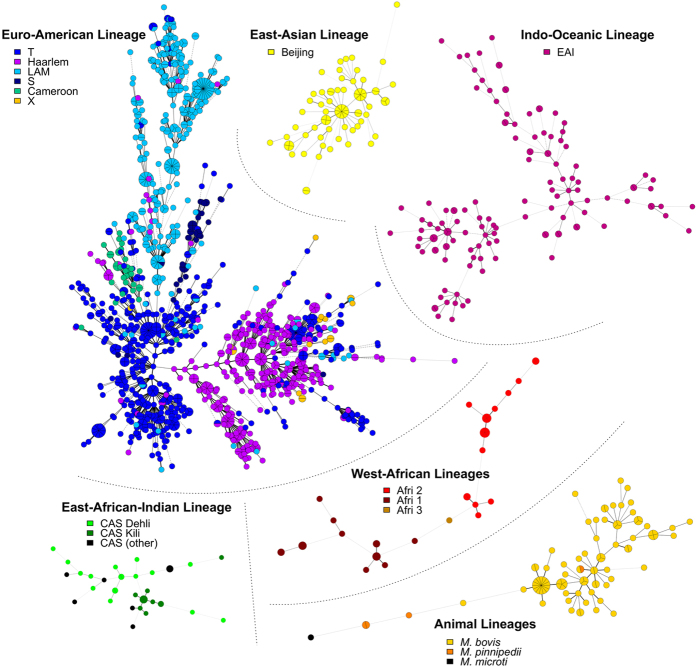
Population structure of MTBC strains isolated from the Rhône-Alpes region of France. Shown are independent MSTrees (one per major lineage) based on 15-loci MIRU-VNTR typing of 1,641 MTBC strains isolated from 2008 to 2014. Lengths of links between nodes are proportional to the number of allelic differences. Larger graph nodes indicate clusters of strains with identical MIRU genotypes. Node colors indicate spoligotype-based families.

Spoligotypes were compared to those of SpolDB4 database^35^, which allowed us to assign isolates into families including AFRI, Beijing, BOV, Cameroon, CAS, Haarlem, LAM, S, T and X^31^, which were then reclassified into 6 major genome sequence- (or genomic deletion-) based lineages, including e.g. the East-African Indian, East Asian, Euro-American, Indo-Oceanic and West African lineages^36^, according to known correspondences^37^. Strains of *M. bovis, M. pinnipedii* and *M. microti* were assigned to the so-called Animal lineage on a same basis. The resulting groupings and correspondences between families (such as Haarlem) and lineages (such as Euro-American) are made apparent in Fig. 3. Strains of the Euro-American lineage were most prevalent (see Supplementary Table S1).

Minimum spanning trees (MSTrees) were constructed based on the 15-loci MIRU-VNTR haplotypes to obtain graphical representations of the relationships between MIRU-VNTR haplotypes within each lineage (Fig. 3). We then investigated how THD analyses correlated with MSTree structures to illustrate how the qualitative and subjective information provided by MStrees is captured by THD in a quantitative and objective fashion. Short- and long-term THD timescales of 20 and 200y, respectively, were used in our analyses. The 200y timescale approximately matched the onset of the Industrial Revolution, previously reported to coincide with the expansion of several MTBC lineages^12^,^13^. The 20y timescale was chosen arbitrarily to reflect transmission over a much shorter period of time, of the same order of magnitude as a human generation. Additionally, 20y can be considered the shortest informative timescale with respect to MIRU-VNTR evolution (using an evolutionary rate of 5 × 10^−4^ change per locus per year, the probability of observing a change among 15 independent markers over 20y is ~14%).

**Figure 4:**
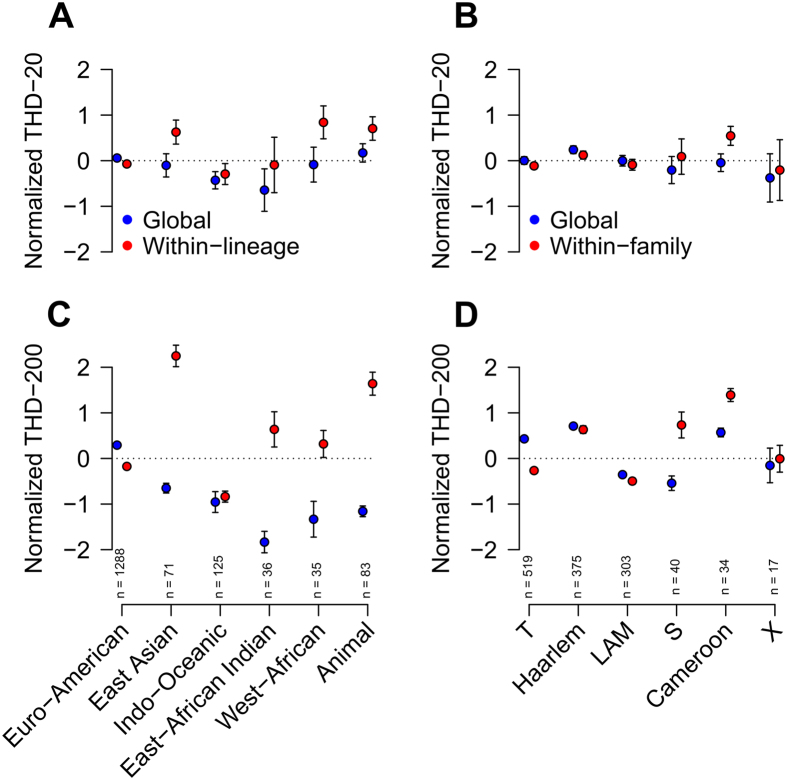
Comparison of time-scaled haplotypic densities (THDs) between MTBC lineages (**A,C**) and spoligotype families within the Euro-American lineage (**B,D**). Short (20y, (**A,B**) and long (200y, (**C,D**) THD timescales were used to reflect short- and long-term evolutionary success, respectively. THDs were computed either with respect to the complete collection of strains (global THD, blue marks) to reflect evolutionary success at the level of the cohort, or independently within each group (within-lineage or -family THDs, red marks) to reflect evolutionary success independent of the global population structure in the collection. Circles denote mean log-THDs; error bars are 95% CI of the mean (not visible when CI is smaller than marker size). Sample size in each group are indicated above the X-axis. Indications of statistical significance were omitted for readability.

To separately investigate diversity and prevalence of lineages and families, THDs were computed either relative to the complete strain collection (hereafter, global THDs) or to each lineage or family, independently (herafter, within-group THDs). In both cases, log-THDs were normalized and summarized as means and 95% confidence intervals of the mean (Fig. 4). Comparisons of global THDs allowed describing the evolutionary success of each group relative to the other groups, taking both prevalence and genetic diversity into account. Within-group THDs ignored the global population structure, mostly reflecting clonality in each group independent of their prevalence or genetic relatedness with other groups. Detailed insights into the relationships of spoligotype family or lineage, timescale and THD measures are provided in Supplementary Fig. S2.

Within-lineage and -family THDs, shown as red markers in Fig. 4, reflected the structural characteristics of the MSTrees inferred from the same groups, shown in Fig. 3. The highest long-term within-lineage THD was found in East-Asian/Beijing strains, consistent with the dense, radial structure of their respective MSTree, suggestive of recent population expansion and diffusion^12^. By contrast, the Indo-Oceanic lineage had the smallest long-term within-lineage THD, consistent with the highly relaxed structure of the MSTree, indicative of genetically diverse strains with few recent transmission events. Between these extreme cases, the Euro-American MSTree was dense, with long branches and no obvious central node. Reflecting this diversity, Euro-American strains had the second smallest long-term within-group THDs (Fig. 4C).

Short-term within-group THDs reflected the distribution of closely related haplotypes in each group. These THDs were comparably high in West-African, animal and East-Asian/Beijing lineages, reflecting the large proportion of strains belonging to clusters of identical MIRU-VNTR haplotypes in these groups (Fig. 3). Of note, identical MIRU-VNTR haplotypes in *M. bovis* did not necessarily reflect recent inter-patient transmission events but also contamination by a common source, namely the Bacillus Calmette Guerin vaccine strain^38^.

Global THDs, shown as blue markers in Fig. 4, take both the clonality and prevalence of group into account. Expectedly, the highly frequent Euro-American lineage had the highest long-term global THD in spite of its less-than-average within-group THD. Thus, this contrast between global- and within-group THDs highlighted the endemic nature of the Euro-American lineage, both prevalent and diversified, in our setting. Analyses at the sublineage level (Fig. 4D) indicated that the Haarlem and T families mostly contributed to the endemicity of the Euro-American lineage. The Cameroon family, although unfrequent, also had a high THD value, consistent with the previously reported success of this clade in Western Africa^39^, from which most patient infected with Cameroon strains originated (n = 17/20, 85%). The East-Asian/Beijing lineage had the second highest long-term global THD in spite of being ranked fourth by decreasing order of prevalence. Interestingly, this lineage also exhibited a high short-term global THD. In line with the radiating MSTree structure observed for this lineage, this pattern indicates that Beijing strains, although neither prevalent or endemic in our setting, exhibit a high degree of clonality suggestive of a recent epidemic success.

Collectively, these analyses identified the Euro-American strains, mostly in the Haarlem and T families, as being part of the endemic background of tuberculosis in our setting. The results also highlight the recent epidemic success of Beijing strains in spite of their low prevalence.

### Factors associated with short- and long-term epidemic success of MTBC strains

Using global THD20 and THD200 as proxies for short- and long-term epidemic success in MTBC strains and their respective lineages, we conducted association studies to identify characteristic features of successful strains and of their infected hosts. Bivariate linear regression analyses detected several such success-associated features (Table 2). Importantly, two of these associations could be considered as positive controls of our analysis. First, smear-positive patients have a well-known higher risk of transmitting disease and of being part of a recent transmission chain, hence their isolates were expected to exhibit higher THD20 values. Second, considering that patients are more likely to harbor strains that are endemic in their region of origin^4^,^5^, isolates from French-native patients were expected to exhibit higher THD200 values. Both associations of THD20 with sputum smear positivity and of THD200 with French-native status had indeed highly significant *P*-values in bivariate analysis. This indicated that THD correctly identified these known and relevant epidemiological processes, in turn suggesting the relevance of this analysis for detecting other associations. Where applicable, we thus examined these associations in more details using stratified analyses and multiple regression models controlling for potential confounders.

Along with sputum smear positivity and pulmonary infection, THD20 correlated with younger age, in line with previous observations that MTBC genotype clustering was more frequent in younger patients^3^. This association was still significant after excluding *M. bovis* strains from the analysis (*P* = 6.7 × 10^−3^), indicating a link with patient-to-patient transmission patterns rather than a bias due to Bacillus Calmette Guerin vaccine strain-related infections in infants. However, separate spline regression curves (see Methods) constructed for French-native and non-native patients (Supplementary Fig. S3) indicated that the association pattern of age with THD20 was specific of French-native patients, as THD20 did not change with age in non-native patients. Surprisingly, the association of student status with smaller THD200 retained its amplitude after controlling for age and French-native status, although not significantly so (coefficient −0.49, *P* = 0.07). Among the 201 patients with known occupation and country of origin, 31 (15.4%) were students of which 24 (77.4%) were French-native, suggesting that lower THD200 in MTBC-infected students was not related to a high proportion of non-French-native students in our cohort. Indeed, when restricting the analysis to French-native patients (n = 225) and controlling for age, student status was still associated with a lower THD200 (coefficient −1.06 compared to employed patients, *P* = 0.019). Collectively, these results suggest that students are more likely to be infected with MTBC strains that do not belong to the endemic background.

MTBC strains involved in a pulmonary infection exhibited larger THD200s both in bivariate analysis (Table 2) and after controlling for age and French-native status (coefficient 0.28, *P* = 3.1 × 10^−3^). The proportion of pulmonary infections varied depending on the continent of origin of the patients (*P* < 10^−5^, Fisher’s exact test), from 64.0% (n = 174/272) in African-born patients to 74.3% (n = 55/74) and 84.3% (n = 210/249) in Asian- and European-born patients, respectively (other continents omitted due to small sample sizes), and on the lineage of the infecting strain (*P* < 10^−5^). This proportion was largest in strains of the East-Asian/Beijing lineage (n = 40/50, 80.0%), followed by the West-African (n = 19/25, 76.0%), Euro-American (n = 522/693, 75.3%), Indo-Oceanic (n = 48/74, 64.9%), East-African Indian (n = 10/20, 50.0%) and animal (n = 15/57, 26.3%) lineages. The association of pulmonary infection with THD200 retained significance after controlling for the continent of origin (*P* = 0.002) but not for the phylogenetic lineage (*P* = 0.93; both models also controlled for age). Hence, the bivariate association of THD200 with pulmonary infection mainly resulted from the association between the two most successful lineages in the long-term, namely the Euro-American and East-Asian/Beijing lineages (Fig. 4C), with high proportions of pulmonary infections compared to other lineages. To determine whether pulmonary infection influenced THD200 at the sub-lineage level, regression models controlled for age and French-native status were constructed for each lineage independently. An independent association was still present between pulmonary infection and THD200 in strains of the East-Asian/Beijing lineage (coefficient 0.76, *P* = 2.9 × 10^−3^) but not of other lineages, which suggested that the ability of MTBC Beijing strains to cause pulmonary infection influenced their long-term epidemic success.

## Discussion

To our knowledge, the THD framework represents the first approach to allow for in-depth joint analysis of epidemic success over time with pathogen- and host-associated factors in highly structured pathogen populations. By applying this approach on a large cohort of TB patients, we identified factors that contributed to the short- or long-term epidemic success of particular strains in a typical low-prevalence, French setting.

Interestingly, associations of bacterial- and host-related factors with epidemic success/THD measures (Table 2) depended on the timescale considered. As a consistent example, short-term THD, hence short-term epidemic success, was associated with positivity of smear sputum, which is well known to impact on patient contagiousness^40^. Sputum positivity, which reflects disease severity^41^,^42^, is thought to be linked to host-related factors, both behavioral or connected to genetic susceptibility^42^,^43^, but perhaps as well to pathogen-related factors^44^. The general causal relationship between long-term THD and epidemic success and pulmonary forms of TB, representing the infectious form of the disease, is also straightforward. More remarkably, the Euro-American and Beijing lineages exhibited both high long-term THD values and rates of pulmonary TB (see Supplementary Fig. S4). Higher rates of pulmonary infection caused by strains of these lineages has been reported previously by Click *et al*. in the US population^45^. This association was reportedly independent of race/ethnicity, HIV status, age and sex, suggesting that it reflected lineage- rather than patient-specific characteristics. Such interpretation is further supported by the striking similarity between per-lineage proportions of pulmonary disease reported by Click *et al*. in their US patient population and those found in our French cohort: 87.0 vs. 80.0%, 86.2 vs. 75.3%, 77.4 vs. 64.9% and 65.7 and 50.0% for the East Asian/Beijing, Euro-American, Indo-Oceanic and East African-Indian lineages, respectively (R^2^ = 0.99, *P* = 0.006). Taken collectively, these results bring additional support to the hypothesis of MTBC lineage-specific adaptations impacting on disease^1^,^44^,^46^,^47^,^48^,^49^, including the ability to generate active pulmonary TB as a major driving force of MTBC population dynamics^44^,^50^.

Some limitations of our study prevented us to test several hypotheses. In particular, the proportion of missing data was high for several possibly important factors, such as the time of arrival in France of non-native patients (Table 1), and individual risk factors for tuberculosis such as HIV infection or other immunological impairments were not available for analysis. Although ignoring these factors is unlikely to have biased our conclusions regarding the relationship of pulmonary tuberculosis and long-term epidemic success of MTBC lineages, their inclusion in models involving short-term THD could have helped refining our association analysis.

Compared to current maximum-likelihood or Bayesian methods for investigating pathogen demography, THD is less computationally demanding due to its simplicity, potentially allowing for the analysis of larger strain collections. This computational efficiency is linked to the absence of an explicit phylogenetic reconstruction and to the choice of the efficient but approximate infinite alleles model (IAM) to calibrate the bandwidth (see Methods). These methodological choices have practical consequences regarding the applicability of THD for future studies. First, due to the absence of an evolutionary model, THD can handle any type of qualitative genetic data that bears phylogenetic information, such as minisatellites or DNA polymorphisms. Second, although the IAM model is reasonably accurate for recent TMRCAs^51^, it does not consider locus homoplasy and tends to underestimate TMRCA when genetic distance increases, as illustrated in Supplementary Fig. S5. Hence, THD analyses should be restricted to relatively recent timescales so that locus homoplasy can be safely ignored. We empirically suggest that the chosen timescale should not yield a median genetic distance greater than one-third of the number of loci (corresponding to a maximal timescale of ≈400y for 15-loci MIRU-VNTR; Supplementary Fig. S5). Finally, one should keep in mind that typing methods of routine use such as 15-loci MIRU-VNTR convey much less information than, e.g., whole genome sequences, and that a large sample size (say, n > 100) is desirable to compensate for the uncertainty inherent to low-resolution data (Fig. 2 and Supplementary Fig. S5).

In summary, our results describe how the interplay of MTBC lineage specificities and host risk factors contribute to the large-scale population dynamics of MTBC in a low-prevalence setting. Analyses focused on longer or shorter timescales confirmed the potential driving forces of the epidemic success of MTBC such as the propensity to cause transmissible, pulmonary disease in the long run and sputum-positive infections in the short run. Such approach could be used more generally to infer the epidemic success of pathogens with widely available typing data, including SNPs, and to reveal relevant associations with factors suspected to influence the course of an epidemic over time.

## Methods

### Timescaled haplotypic density

We consider the problem of using kernel density estimation to assign a measure of density to a haplotype, represented as a vector of markers, relative to a set of other haplotypes. After providing the required definitions, we briefly expose the kernel function, the computation of the bandwith based on a timescale parameter, and we provide a synthetic overview of THD computation. Source code of the software implementation of these methods for the R platform is available in the Supplementary Note.

Let *X* be a sample of *n* haplotypes defined over *m* markers, represented as an (*n*×*m*) data matrix, and let *y* be a haplotype of interest not in *X*. For each haplotype *x*_*i*_ in *X*, let *h*_*i*_ be the genetic distance from *y* to *x*_*i*_, i.e. the number of differences between *x*_*i*_ and *y*. A genetic distance *h* is associated with a kernel density (formally, a probability) *k(h*|*b,m*) under the truncated geometric distribution with bandwidth *b* (formally, the failure probability of a Bernoulli trial) and truncation limit *m*. This distribution has probability mass function  
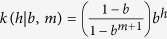
. The bandwidth *b* is a real number between 0 and 1. The density associated with a given distance *h* is proportional to *b*^*h*^, which illustrates how the bandwidth controls the influence of *h* on the density: for each additional difference between *y* and *x*, the density is multiplied by *b*. Reducing *b*, thus, accelerates the decrease of the density for larger numbers of differences. Finally, the haplotypic density *K(y*|*X,b,m*) of *y* with respect to *X* is the average of the *n* densities associated with the distances from *y* to each *x*_*i*_ in *X*,


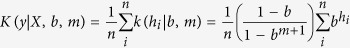


Because *b* is a dimensionless constant, its choice is not intuitive. To circumvent this issue, we exploit the existence of a one-to-one relationship between genetic distance *h* and the maximum-likelihood estimate of the TMRCA *t* under the infinite alleles model (IAM)^51^, which assumes that the *m* haplotype markers lie on a non-recombining DNA segment, that they evolve independently with a common evolutionary rate *μ*, and that at most one change per marker occurred in both lineages since their MRCA. Assuming that *μ* is known, the IAM model allows to replace the bandwidth with a more intuitive timescale parameter *t*_50_, or tMRCA50, which is the TMRCA such that haplotypes with shorter TMRCAs account for 50% of the density. Practically, we solve the IAM model relation *t* = log[*m*/(*m*−*h*)]/2*μ* for *h* to obtain *h* = (1−*e*^−2*μt*^)*m*. This relation allows to associate a (possibly non-integer-valued) distance *h*_50_ with the chosen timescale *t*_50_. From the definition of *t*_50_, it follows that *h*_50_ is the median of a truncated geometric distribution whose bandwith b_*_ must be determined. From the cumulative probability function of the truncated geometric distribution with parameters *b* and *m, P(H* ≤ *h*|*b, m*) = 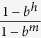, it follows that if *h*_50_ is the median of the continuous form of the distribution with bandwidth b_*_ then b_*_ must satisfy 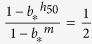, an equation which we can solve for b_*_ numerically (as no closed-form solution exists) using a root-finding algorithm over the [0,1] interval.

THD computation steps can be summarized as follows: (i) determine parameters *m* (number of markers), *μ* (evolutionary rate) and *t*_50_ (timescale); (ii) associate the timescale with a median distance *h*_50_; (iii) determine the corresponding bandwidth b_*_; and iv) for each haplotype of interest, compute THD as the average kernel density under the truncated geometric distribution with bandwidth b_*_ and truncation limit 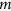.

### Summarizing, scaling and normalizing THD

Because THDs are probabilities, aggregate statistics for groups of isolates should use products to represent the joint likelihood of isolates in the group. As a consequence, we use geometric means rather than arithmetic means, and transform THDs to logarithms before inclusion in linear models. Because THD estimates are inversely proportional to the number of loci *m* and sentitive to the timescale we propose two modifications to ease comparability. First, THDs can be multiplied by the number of loci. These scaled THDs are invariant relative to *m* (Fig. 2 and Supplementary Fig. S1), which might facilitate comparison between THDs with similar timescale but obtained with different methods, e.g. minisatellite typing vs. DNA sequencing. Second, log-THDs can be centered and scaled relative to the population under study. These normalized THDs are multiples of standard deviations from the mean of the population, similar to Z-scores. They are not comparable across studies, as they depend on a given population, but they can be compared between different timescales.

### Simulation experiments

Simulation of minisatellite-based haplotypes, evolving under the stepwise mutation model using a continuous-time sequential Markov coalescent approximation, were conducted by means of Fastsimcoal2 software^22^. Scenario parameters were set as indicated in text. Simulated haplotypes were imported into the R software environment for THD computation and further analyses.

### Ethics statement

This retrospective, cross-sectional, observational multicentric study was approved by the Comité de Protection des Personnes Sud-Est IV under no. DC-2011-1306. Written consent of participants was not obtained, in accordance with French regulations, due to anymous treatment of data and the non-interventional nature of the study.

### Patient population and collection of data

Patients were identified retrospectively from the surveillance database of the ORAM, a regional collaborative surveillance system active since 2005 whose participants include: i) the microbiology laboratories of the three university hospitals of the Rhône-Alpes region, namely Lyon, Grenoble and Saint-Etienne, as well as other microbiology laboratories in charge of tuberculosis diagnosis; ii) the Agence Régionale de Santé (ARS) to which all TB diagnoses are notified by practitioneers as part of the French programme for tuberculosis surveillance; and iii) the Centre de Lutte Anti-Tuberculeuse (CLAT) which is in charge of the identification of contact cases and of the long-term follow-up of tuberculosis patients after hospital discharge. MTBC strains isolated by the participating laboratories are routinely referred to reference laboratories for molecular typing, including spoligotyping since 2005 and 15-loci MIRU-VNTR typing since 2008. Typing methods were consistent in all ORAM laboratories. Spoligotyping was performed as described elsewhere^29^. Spoligotypes were compared to the SpolDB4 database of Institut Pasteur and assigned to lineages and sublineages^35^.

Patients were eligible if the tuberculosis diagnosis was notified to the ARS between 2008 and 2014 and if their infecting strain had been isolated and typed (n = 1,746). Patients whose MTBC strain had ambiguous MIRU-VNTR profile (i.e., undefined number of repeats at any of the 15 loci; n = 105) were excluded. Demographic data extracted from the database included gender, age at the time of diagnosis, year of isolation of the MTBC strain, country of birth, occupation (employed, unemployed, student or retired), and collective dwelling (including nursing home, group home, prison and refugee camp). Disease-related data included disease location, sputum smear positivity and phenotypic rifampin and isoniazide resistance. Disease was classified as pulmonary or exclusively extra-pulmonary. Patients with exclusively extra-pulmonary disease were those with at least 3 sputum samples with negative MTBC culture result. If <3 sputum samples were taken, disease location was considered unknown (Table 1).

### Population structure analysis

MSTrees were computed based on the 15-loci MIRU-VNTR haplotypes using BioNumerics 7.5 (Applied Maths, St Martens-Latem, Belgium). An MSTree is a connected undirected graph selected to minimize the sum of marker differences over all links between haplotypes, enabling the graphical representation of quantitative relationships between MIRU-VNTR haplotypes. Independent MSTrees, one per major lineage, were constructed.

### Statistical analysis

Association studies of socio-demographic, disease-related and microbiological parameters with THD measures were conducted by means of multiple linear regression models with log-THD as the response variable. Control for confounding was achieved by including potential confounders, indicated in text as appropriate, as covariates. Acceptability of linear regression assumptions was assessed by visual inspection of residual distributions and quantile-quantile plots. In line with the exploratory nature of the study, no *P*-value correction for multiple testing was applied. The significance threshold was set at 0.05 for all tests. Spline regression curves based on cubic spline interpolation with automatic selection of the smoothing parameter were used to visualize possible non-linear relationships between variables. All computations were performed using R software version 3.0.1 Good Sport (The R Foundation for Statistical Computing, Vienna, Austria).

## Additional Information

**How to cite this article:** Rasigade, J.-P. *et al*. Strain-specific estimation of epidemic success provides insights into the transmission dynamics of tuberculosis. *Sci. Rep.* **7**, 45326; doi: 10.1038/srep45326 (2017).

**Publisher's note:** Springer Nature remains neutral with regard to jurisdictional claims in published maps and institutional affiliations.

## Supplementary Material

Supplementary Information

Supplementary Source Code

Supplementary Dataset 1

## Figures and Tables

**Table 1:** Socio-demographic and disease-related characteristics of 1,641 MTBC-infected patients from the French Rhône-Alpes region, 2008–2014.

Factor^a^		Cases with available data (%)
Median age at diagnosis [IQR]	48 [31–72]	1636 (99.7)
Male sex (%)	947 (57.7)	1640 (99.9)
French-native (%)	225 (32.9)	683 (41.6)
Median time in France before diagnosis for non-native patients [IQR]	5 [0–15]^b^	156 (36.4)
Collective dwelling (%)	65 (24.3)	268 (16.3)
Occupation (%)	—	227 (13.8)
*Employed*	69 (30.4)	—
*Retired*	76 (33.5)	—
*Student*	36 (15.9)	—
*Unemployed*	46 (20.3)	—
Pulmonary infection (%)	656 (71.2)	921 (56.1)
AFB-positive sputum (%)	288 (44.4)	648 (39.5)
Rifampicin resistance (%)	32 (3.8)	836 (50.1)
Isoniazid resistance (%)	69 (10.1)	679 (41.4)
Multidrug resistance (%)	25 (3.7)	679 (41.4)

^a^Numbers of patients or strains (%) unless specified otherwise; IQR, interquartile range.

**Table 2:** Socio-demographic and disease-related factors associated with short- and long-term time-scaled haplotypic densities (THD) in MTBC-infected patients.

Factor	Short-term THD (20y time-scale)		Long-term THD (200y time-scale)	
	Coeff. (95% CI)^a^	*P*-value	Coeff. (95% CI)	*P*-value
Age at diagnosis (per 10 years)	**−0.04 (−0.06, −0.02)**	**1.8 × 10**^**−4**^	**0.08 (0.06, 0.01)**	**7.8 × 10**^**−16**^
Male sex	0.02 (−0.08, 0.12)	0.73	−0.06 (−0.16, 0.04)	0.21
French-native	0.15 (−0.02, 0.31)	0.08	**0.39 (0.23, 0.55)**	**2.1 × 10**^**−6**^
No. of years in France before diagnosis (non-native patients)	0.00 (−0.01, 0.01)	0.80	0.00 (−0.01, 0.01)	0.92
Collective dwelling	0.07 (−0.21, 0.36)	0.61	0.17 (−0.12, 0.46)	0.26
Occupation^b^	—	0.36	—	**1.6 × 10**^**−3**^
* Employed*	Reference	—	Reference	—
* Retired*	−0.21 (−0.56, 0.15)	0.25	**0.40 (0.05, 0.75)**	**2.6 × 10**^**−2**^
* Student*	−0.29 (−0.73, 0.15)	0.19	**−0.44 (−0.87, −0.01)**	**4.7 × 10**^**−2**^
* Unemployed*	0.04 (−0.36, 0.45)	0.83	0.06 (−0.34, 0.46)	0.78
Pulmonary infection	**0.17 (0.03, 0.3)**	**2.0 × 10**^**−2**^	**0.24 (0.09, 0.38)**	**1.2 × 10**^**−3**^
AFB-positive sputum	**0.34 (0.18, 0.50)**	**2.0 × 10**^**−5**^	0.15 (−0.01, 0.30)	0.06
Rifampicin resistance	0.14 (−0.22, 0.50)	0.44	−0.13 (−0.49, 0.23)	0.47
Isoniazid resistance	−0.01 (−0.26, 0.25)	0.96	**−0.33 (−0.59, −0.08)**	**9.4 × 10**^**−3**^
Multidrug resistance	0.20 (−0.21, 0.61)	0.33	−0.1 (−0.50, 0.31)	0.64

^a^Coefficients of linear regression of normalized log-THD, expressed as multiple of standard deviation. Significant coefficients and *P-*values (*t*-test) highlighted in bold.

^b^Reported are the model-wise *P*-value of multiple regression model (*F*-test) and category-specific coefficients taking the employed category as reference.
