# Supplementary material for: Strain-specific estimation of epidemic success provides insights into the transmission dynamics of tuberculosis
Source: Sci Rep. 2017 Mar 28;7:45326. doi: 10.1038/srep45326 (PMC5368603; doi:10.1038/srep45326)
Supplement: Supplementary Information [file srep45326-s1.pdf]

Strain-specific estimation of epidemic success provides insights into the transmission dynamics of tuberculosis

Jean-Philippe Rasigade, Maxime Barbier, Oana Dumitrescu, Catherine Pichat, Gérard Carret, Anne-Sophie Ronnaux-Baron, Ghislaine Blasquez, Christine Godin-Benhaim, Sandrine Boisset, Anne Carricajo, Véronique Jacomo, Isabelle Fredenucci, Michèle Pérouse de Montclos, Jean-Pierre Flandrois, Florence Ader, Philip Supply, Gérard Lina & Thierry Wirth

Supplemental Information File

Supplementary Table S1. Distribution of major MTBC lineages and families in a French cohort, 2008-2014.

Lineage	Family	No. of isolates (%)
Euro-American	T	519 (31.6)
	Haarlem	375 (22.9)
	LAM	303 (18.5)
	Total	1,288 (78.6)
Indo-Oceanic		125 (7.6)
Animal[a]		83 (5.1)
East-Asian / Beijing		71 (4.3)
East-African Indian		36 (2.2)
West-African		35 (2.1)
Non-assignable		3 (0.2)
Total		1,641 (100)

[a]Including *M. bovis*, n = 78, *M. pinnipedii*, n = 4, and *M. microti*, n = 1.

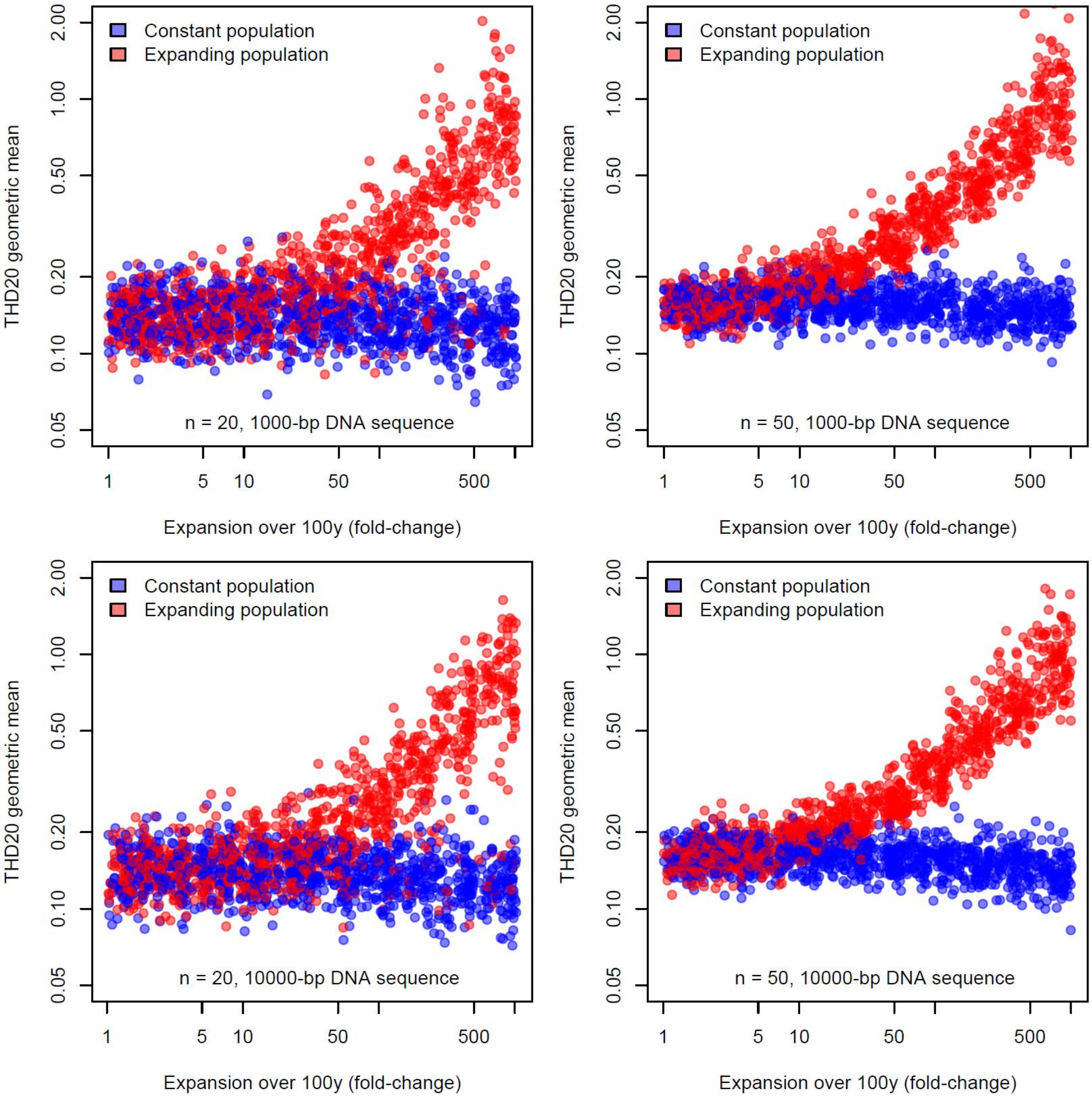

Supplementary Figure S1. Timescaled haplotypic density (THD) of simulated constant-size and expanding populations. Markers represent scaled THD geometric means for 1,000 simulated metapopulations per panel, each comprising of a basal population with constant effective size (blue) and an epidemic population expanding with exponential growth over 100y (red) with varying expansion fold-change (X-axis), sample size per population and DNA sequence length. The evolution model for the DNA sequence involved a per-nucleotide substitution rate of 10^{-7} change per year and no recombination.

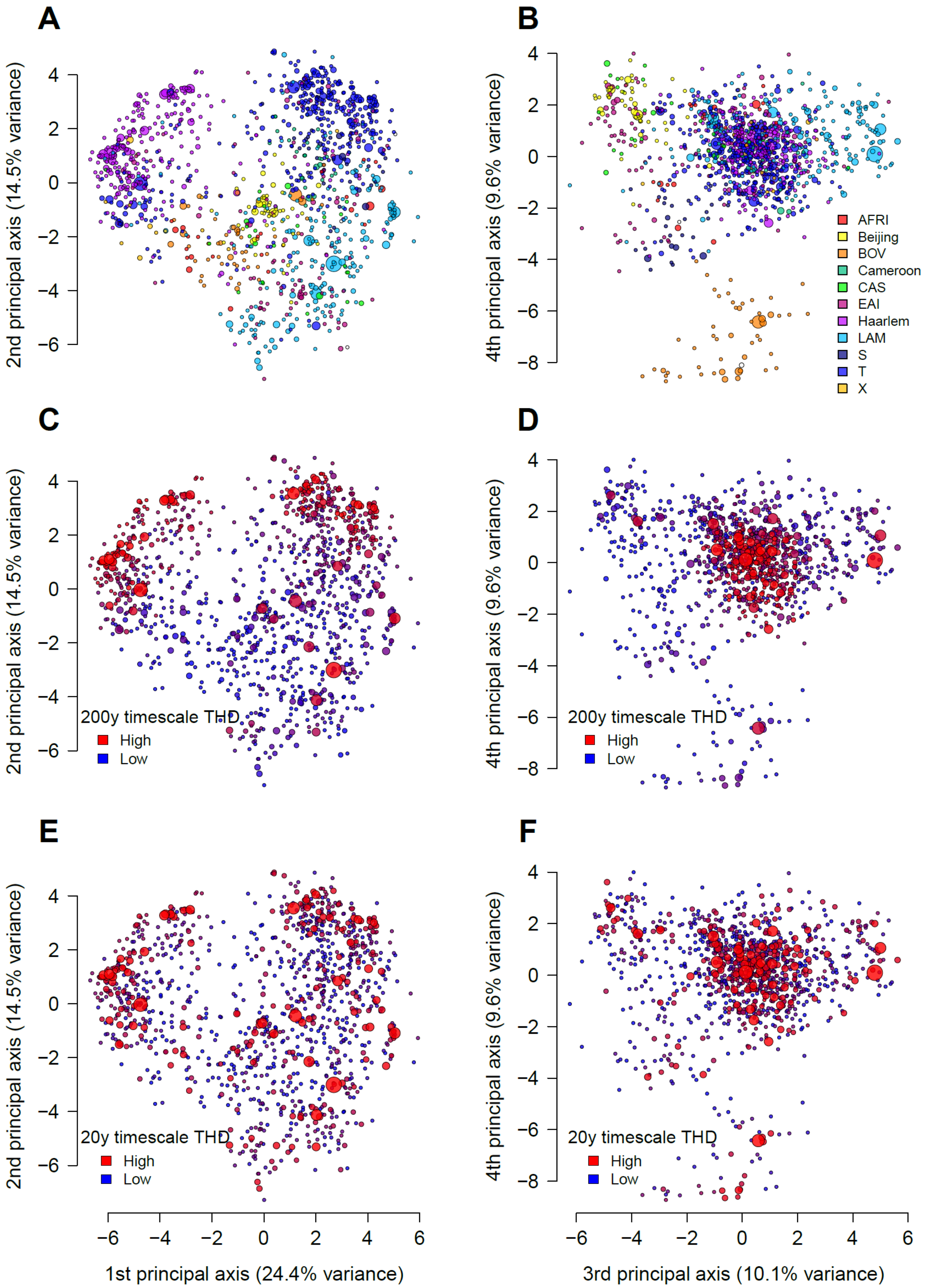
A
B
C
D
E
F
2nd principal axis (14.5% variance)
4th principal axis (9.6% variance)
1st principal axis (24.4% variance)
3rd principal axis (10.1% variance)
AFRI
Beijing
BOV
Cameroon
CAS
EAI
Haarlem
LAM
S
T
X
200y timescale THD
20y timescale THD
High
Low

Supplementary Figure S2. Comparison of THD measures with short and long timescales in a multidimensional scaling (MDS) projection of 1,641 MIRU-VNTR haplotypes. Distances between 15-loci MIRU-VNTR haplotypes were defined as the number of marker differences. Shown are the first two MDS planes resulting from the projection of the distance matrix, with the first plane in left panels (A, C and E) and the second plane in right panels (B, D and F). Marker sizes are proportional to the number of identical MIRU-VNTR haplotypes so that larger markers indicate clustered isolates. In panels A and B, marker color indicates the spoligotype-derived family of the corresponding isolate, illustrating the high prevalence of Haarlem and T isolates. In panels C and D, marker color indicates normalized THD with long timescale (200y). Haplotypes in dense regions (upper-left and –right areas of the first MDS plane and central area of the second MDS plane) were assigned elevated THD measures even when the number of identical haplotypes was low (small markers), while haplotypes in low-density regions were assigned low THD values even when several identical haplotypes were clustered (larger markers). A shorter timescale of 20y was used in panels E and F. Compared to the 200y timescale, clusters of identical haplotypes were assigned higher THD measures even when lying in low-density areas, while non-clustered haplotypes were assigned lower THD measures even when lying in high-density areas.

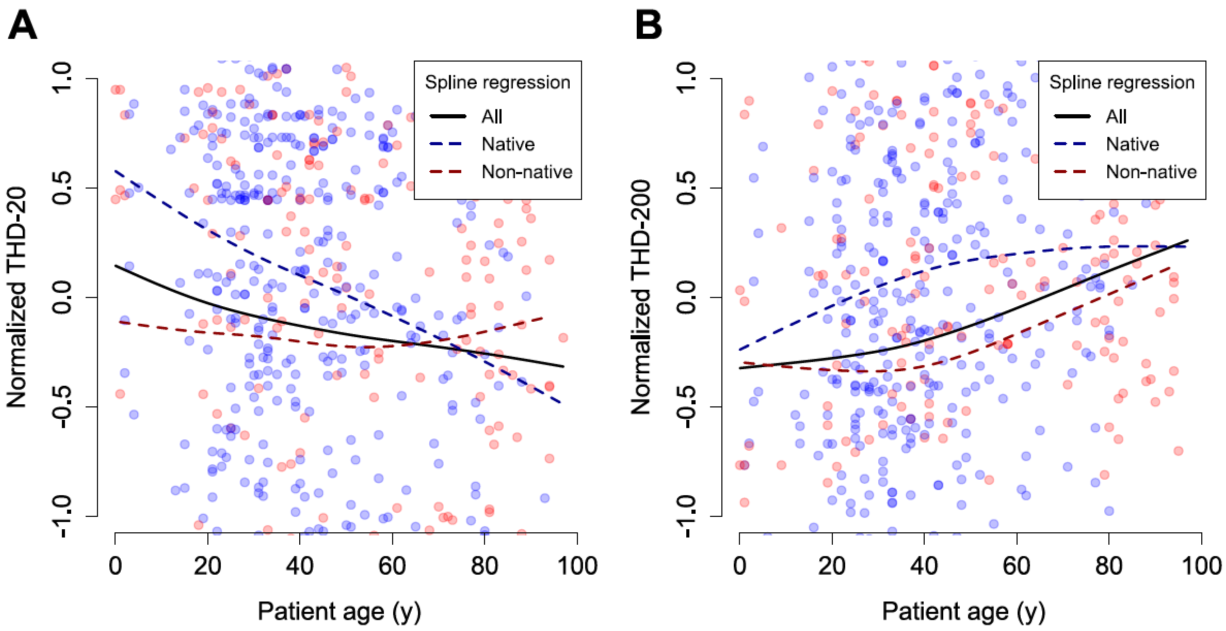

Supplementary Figure S3. Variations of short- and long-term time-scaled haplotypic densities (THD) with patient age and French-native status in 1,641 MTBC-infected patients. Shown are scatterplots of THD with respect to age in French-native and non-native patients (blue and red dots, respectively), and spline regression curves of THD on age for all patients and native and non-native subsets. Y-axis clipped to the center of THD distribution for readability. A 20y short-term THD timescale (THD-20) was used to reflect MTBC epidemicity (A). THD-20 decreased with age in the cohort, however this decrease was observed in native patients, not non-native patients. THD-200 was used to reflect endemicity (B). The cohort-wise increase of endemicity with age was still observed in both native and non-native subgroups but exhibited different patterns.

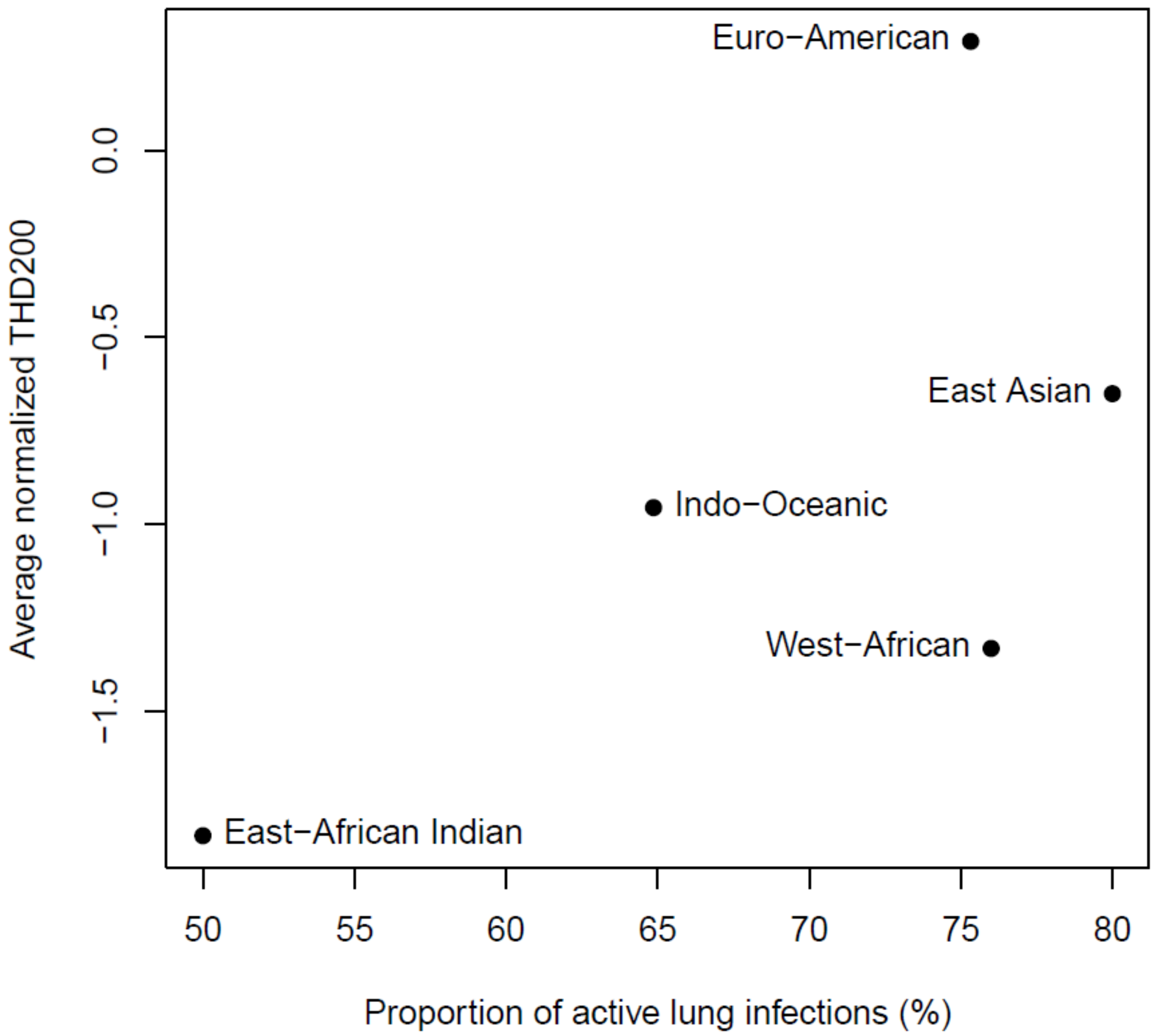

Supplementary Figure S4. Scatterplot of the average timescaled haplotypic density with 200y timescale as a function of the proportion of active lung infections in five major MTBC lineages. Animal lineage was excluded due to the specific transmission routes of its representatives. The Euro-American and East-Asian/Beijing lineages had the highest average THD200 and caused the largest proportions of active, transmissible pulmonary infections.

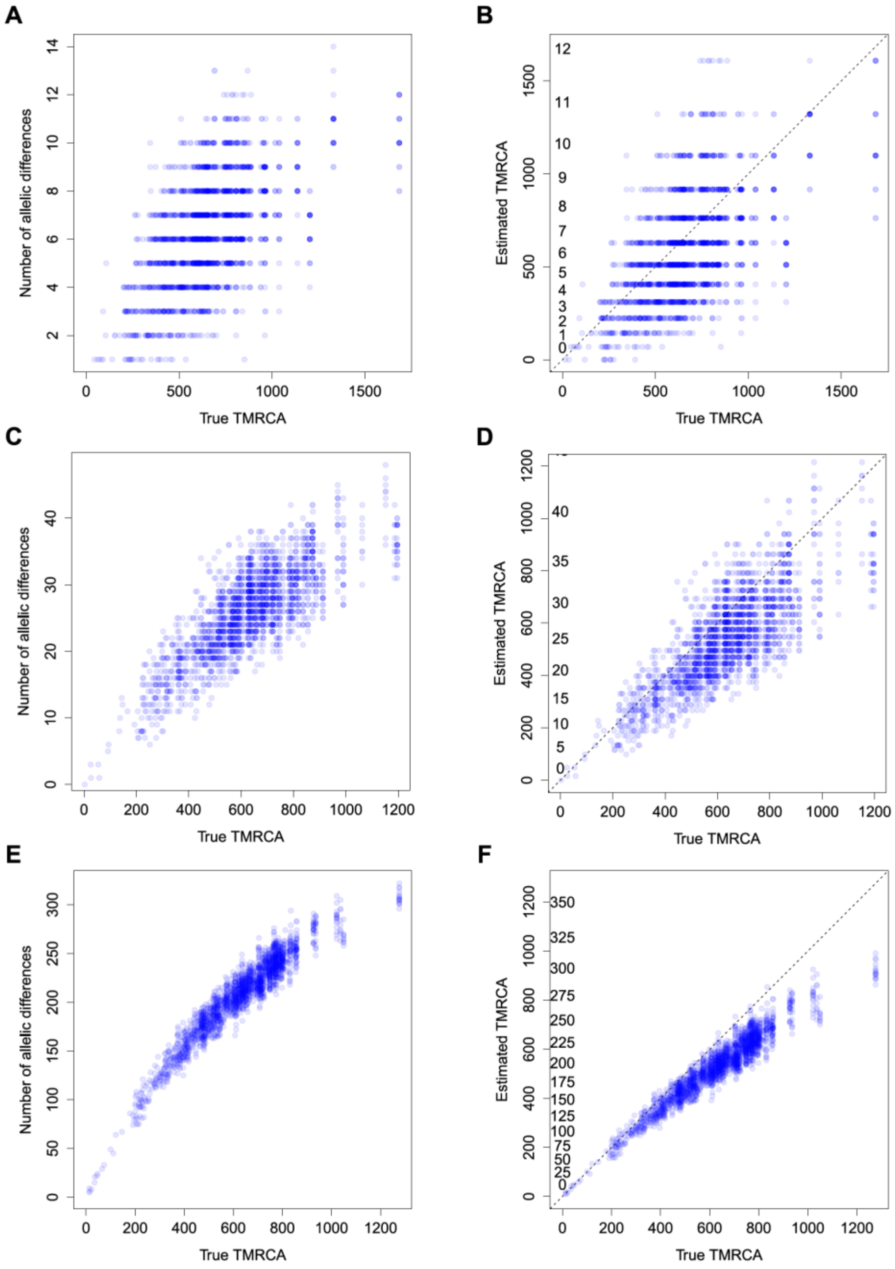

Supplementary Figure S5. Accuracy of the infinite alleles model (IAM) for maximum-likelihood estimation of TMRCA based on genetic distance. Left panels (A, C, E) show the distribution of the genetic distance as a function of the true TMRCA in a simulated population. Note the concave shape of the curve corresponding to homoplasy accumulation for long TMRCAs. Right panels (A, D, F) are scatterplot of IAM estimates of TMRCA against the true values. The corresponding genetic distance is indicated on the right of the Y-axis. The number *m* of markers per haplotype were *m* = 15 (A,B), 64 (C, D) and 512 (E, F).

Supplementary Methods.

Cohort exhaustivity estimation. To estimate the proportion of TB cases from the Rhône-Alpes region of France that were included in our cohort, we combined data from the active TB surveillance program and from a previous study of the proportion of TB cases notified to the surveillance network. In 2010, 433 TB diagnoses were notified (URL: http://www.cdhs.fr/qui-sommes-nous/les-centres-de-sante-et-de-prevention?id=9; accessed Feb 26th, 2017), of which 330 (76.2%) were included in our cohort. A previous study estimated that 73% of all TB cases diagnosed in the Rhône-Alpes region were captured by the notification program (URL: http://invs.santepubliquefrance.fr/content/download/132429/473714/version/1/file/RA_2015_Cire_Rhone-Alpes.pdf; accessed Feb 26th, 2017). Hence, approximately 433/0.73=593 TB cases were diagnosed in the Rhône-Alpes region in 2010, of which 330 (55.6%) were included in our cohort.
